# Supplementary material for: Molecular Iodine/Cyclophosphamide Synergism on Chemoresistant Neuroblastoma Models
Source: Int J Mol Sci. 2021 Aug 19;22(16):8936. doi: 10.3390/ijms22168936 (PMC8396562; doi:10.3390/ijms22168936)
Supplement: Supplementary file 1 [file ijms-22-08936-s001.zip › ijms-1317441-supplementary.pdf]

Supplementary Material: Figure S1 and Figure S2

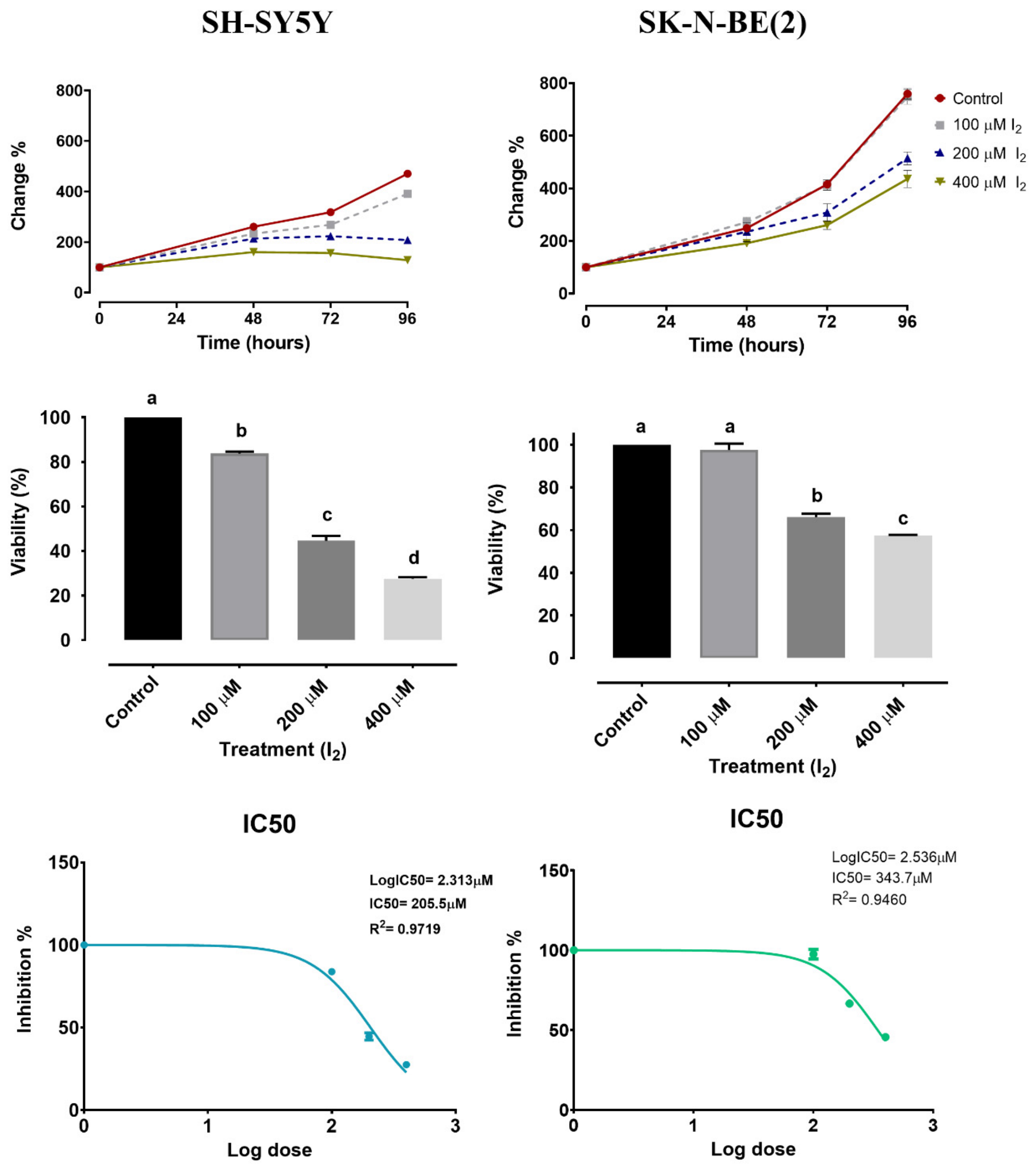

Figure S1. Viability response (% change) and IC50 of SH-SY5Y and SK-N-BE(2) cells to different concentrations of molecular iodine. Different letters denote statistical differences per group ($p < 0.05$).

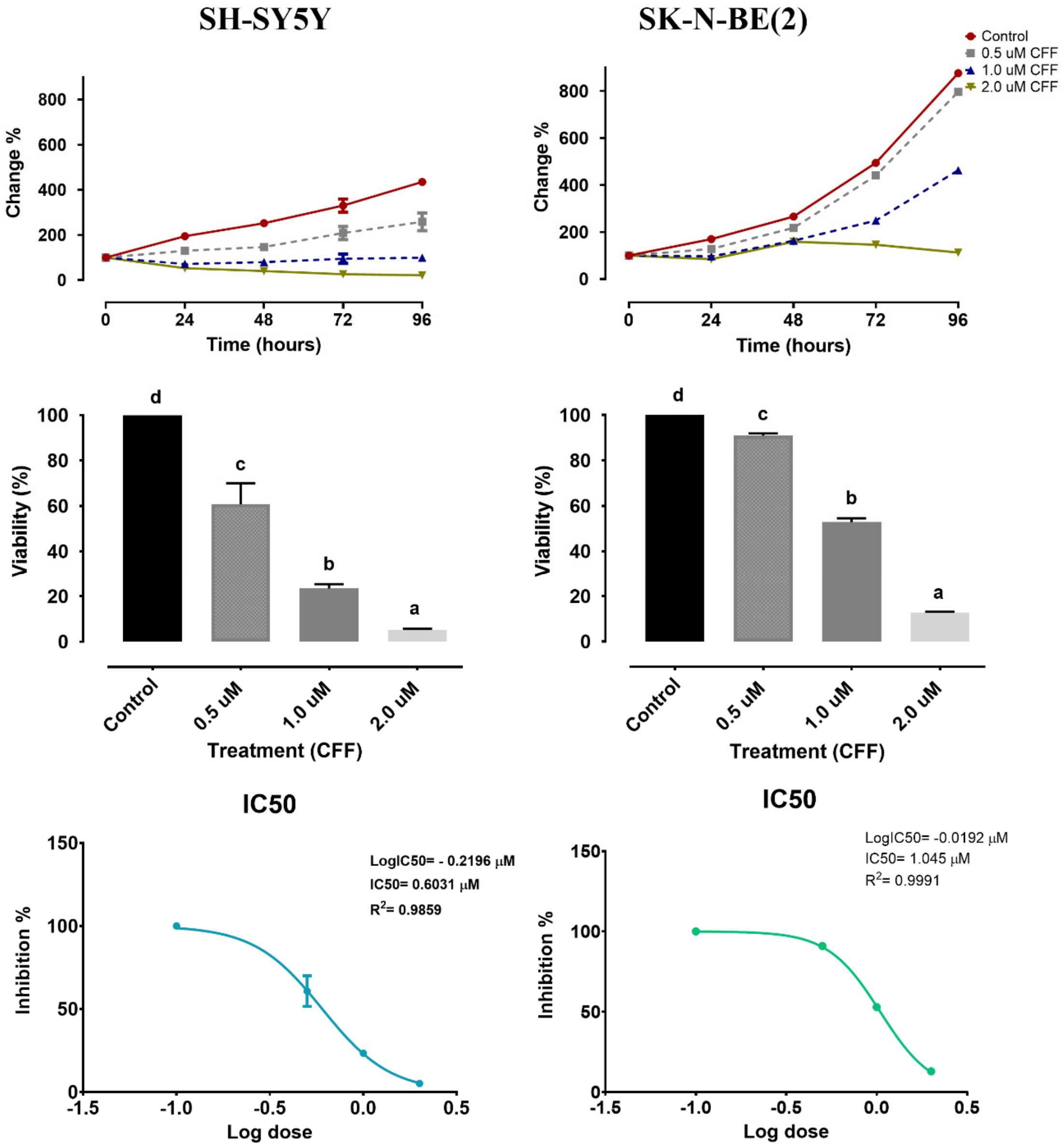

Figure S2. Viability response (% change) and IC50 of SH-SY5Y and SK-N-BE(2) cells to different concentrations of cyclophosphamide. Different letters denote statistical differences per group ($p < 0.05$).
